# Management of Neuroendocrine Breast Carcinoma (NEBC): Review of Literature

**DOI:** 10.3389/or.2024.12114

**Published:** 2024-02-09

**Authors:** A. Mohamed, J. Zeidalkilani, S. L. Asa, M. Trybula, Alberto J. Montero

**Affiliations:** ^1^ Division of Hematology and Medical Oncology, University Hospitals Seidman Cancer Center, Cleveland, OH, United States; ^2^ Department of Medicine, University Hospitals Seidman Cancer Center, Cleveland, OH, United States; ^3^ Department of Pathology, University Hospitals Seidman Cancer Center, Case Comprehensive Cancer Center, Case Western Reserve University, Cleveland, OH, United States

**Keywords:** management, breast, neuroendocrine, carcinoma, review

## Abstract

Extra pulmonary high-grade poorly differentiated neuroendocrine carcinomas (EP-NECs) are rare tumors that usually arise in the gastrointestinal and genitourinary tracts. Primary neuroendocrine carcinoma of the breast (NEBC) is extremely rare, representing less than 0.1% of all breast cancers and less than 1% of neuroendocrine neoplasms. Consequently, they can be misdiagnosed as other types of breast cancer, however, proper immunohistochemical (IHC) studies can assist with making the correct diagnosis. Management of NEBC can be challenging given the paucity of evidence-based literature and should not routinely follow the therapeutic guidelines of other breast cancers. In this article, we review the current literature regarding the management of NEBC.

## Introduction

Although neuroendocrine cells are widely distributed throughout the human body, neuroendocrine neoplasms (NENs) are uncommon. There are multiple definitions, classifications, and nomenclatures that have been used for NENs. However, over the past two decades, NEN nomenclature has evolved to divide them into two major categories: well-differentiated neuroendocrine tumors (NETs) and poorly differentiated neuroendocrine carcinomas (NECs). Primary NECs of the breast are rare tumors (NEBC), with an incidence rate of only 0.3%–0.5%, accounting for 0.1%–5% of breast cancers and less than 1% of neuroendocrine neoplasms [1]. The difference in reported prevalence rates is due to the lack of uniform histological and immunohistochemical (IHC) diagnostic criteria and usually the IHC neuroendocrine markers are not routinely used in the diagnosis of breast cancer. The first case of neuroendocrine differentiation in a breast carcinoma was observed in 1963 by Feyrter and Hartmann. It was not until 1977 when the first case of primary NEBC was reported by Cubila and Woodruff who provided clinical and histological classification for this rare subtype of breast cancer [2, 3]. Because of their low incidence, current knowledge of this tumor is scarce and based on a limited number of anecdotal case reports or small retrospective series. Therefore, their definition, pathological diagnosis, and management remain controversial in the current literature. In this review, we summarized the current evidence about histopathological, immunohistochemical features, as well as management and prognosis of NEBC.

## Histopathology

The 2003 World Health Organization (WHO) breast cancer classification defined NECB as an independent histological entity with immunoreactivity for neuroendocrine markers (chromogranin, synaptophysin, INSM1) in more than 50% of the tumor cells. This WHO classification excluded from their definition breast carcinomas with focal neuroendocrine expression [4, 5]. The classification was later revised in 2012 with two major changes, including the removal of the 50% cut-off value for IHC marker expression, and the categorization of breast neuroendocrine neoplasms (NENs) into three major groups: well differentiated NET, poorly differentiated NEBC/small cell carcinoma, and breast carcinoma with NE features determined by IHC. The latter category included two entities: breast carcinoma of no special type (NST), and special type such as hypercellular mucinous carcinoma or solid papillary carcinoma [6]. With the challenges involved in distinguishing between NEBC and breast carcinoma with NE features, the WHO in 2019 then reclassified breast NENs into either well differentiated NET or poorly differentiated NEBC, which included both small cell NEC and large cell NEC [7]. The NEBC-small cell carcinoma subtype can also be misdiagnosed as lobular carcinoma; however, E-cadherin labeling may help to distinguish between the two tumors. Additionally, hormone receptor (ER and PR) expression is typically observed in NECB [8]. Finally, the differential diagnosis for breast metastasis of a primary NET of another origin (esp. gastrointestinal) should always be excluded by proper IHC.

## Clinical Presentation

The typical median age for NEBC is 60–70 years of age, with a strong female predominance [9]. Clinically, there are no specific or notable endocrine signs or symptoms with regard to NEBC. While well-differentiated NETs have the ability to secrete hormones, poorly differentiated NETs are exclusively non-functional tumors. Limited previous data indicated that NECB can be sporadically present with endocrine syndromes related to ectopic production adrenocorticotropic hormone, norepinephrine, or calcitonin. These data did not distinguish between different subtypes of breast NENs and it is likely that the majority of these are related to well-differentiated NETs and not poorly differentiated NEC [10–12]. The main presentations for NEBC are similar to invasive breast ductal cancers (IBD), i.e., presenting with a palpable breast mass, bloody nipple discharge, skin ulceration or retraction, and axillary lymph nodes enlargement. Most NEBC patients present with metastatic disease at diagnosis, which is due to the aggressive behavior of NECB.

## Diagnostic Evaluation

**FIGURE 1:**
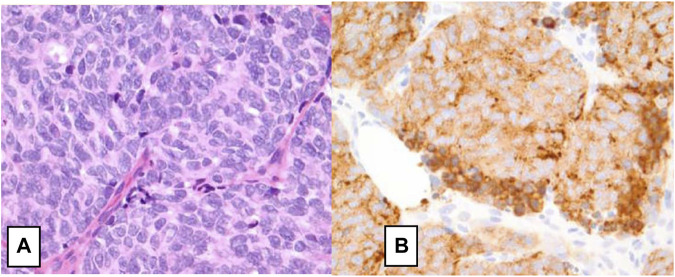
Small cell neuroendocrine carcinoma of the breast. **(A)** H&E staining on high power field (×40) showing neoplastic cells with nuclear pleomorphism, nuclear molding, mitotic figures, hyperchromatic nuclei, minimal cytoplasm, and indistinct nuclei consistent. **(B)** Immunohistochemical staining positive for synaptophysin [14].

**FIGURE 2:**
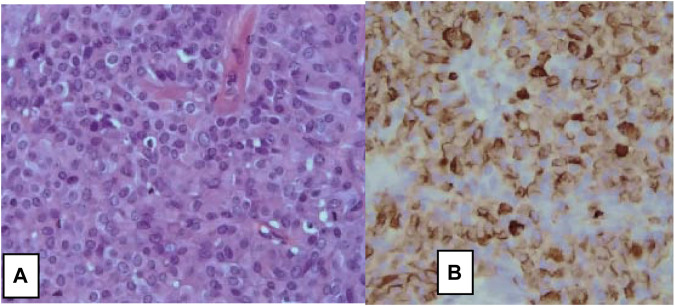
Large cells neuroendocrine carcinoma of the breast. **(A)** H&E stain, ×10, and **(B)** on immunohistochemistry, tumor cells show diffuse positive stain for Chromogranin A (×10) [6].

Diagnostic evaluation for NECB starts with mammography, ultrasonography, and core needle biopsy. Fine Needle Aspiration (FNA) is inadequate for the diagnosis of NECB as they have similar cytological and morphological characteristics seen in other breast tumors such as invasive ductal carcinoma or intraductal papilloma. Therefore, imaging-guided core needle biopsy with IHC staining of neuroendocrine markers is needed for definitive diagnosis of NECB. As most of NEC originate from the lung and gastrointestinal tract, it is additionally important to exclude metastatic tumors from other primary NE sites, and other differentials such as Merkel cell or melanoma before making the diagnosis of primary NECB. The existence of ductal carcinoma *in situ* provides a valid clue to the primary nature of the tumor [13]. Histologic subtypes of NECB includes small cell NEC, large cell NEC, and mixed neuroendocrine/non-neuroendocrine neoplasm of the breast (Br-MINEN) [6, 14] (Table 1) (Figures 1, 2). Small cell NEC is the most common NEBC subtype and, microscopically, it is composed of diffuse proliferation of neoplastic cells with densely packed, small, dark hyperchromatic nuclei, scant cytoplasm with poorly defined cytoplasmic boundaries, and high nuclear/cytoplasmic ratio. By contrast, large cell NEC is an extremely rare NECB subtype which is histologically characterized by large highly pleomorphic nuclei with coarse chromatin and moderate abundant clear or granular cytoplasm and can be misdiagnosed with high grade breast NETs. The specific information about Br-MiNEN is scarce, with most of the published cases including the edition of the WHO classification lacking the clinicopathological studies on this separate identity. Therefore, the criteria for Br-MiNEN diagnosis are adopted from digestive MiNENs. Br-MiNEN contains malignant non-neuroendocrine epithelial components, including adenocarcinoma and more than 30% poorly differentiated NEC components. Combined adenocarcinoma with only IHC expression of neuroendocrine markers without neuroendocrine morphology should not be interpreted as pure Br-MiNEN. For this reason, it is critical to distinguish real Br-MiNEN from carcinomas of the breast with NE and non-neuroendocrine differentiation.

**TABLE 1:** Histologic subtypes of breast neuroendocrine neoplasms (NENs).

Differentiation	Grade	Ki67/MI	Subgroups
Well differentiated neuroendocrine tumor (NET)	G1	Ki67 < 3%	
		MI < 2 mitoses/10HPF	
	G2	Ki67 3%–20%	
		MI 2–20 mitoses/10HPF	
	G3	Ki67 > 20%	
		MI > 20 mitoses/10HPF	
Poorly differentiated neuroendocrine carcinoma (NEC)	G3	Ki67 > 20%	Small cell NEC
		MI > 20 mitoses/10HPF	Large cell NEC
Breast mixed neuroendocrine-non-neuroendocrine (Br-MINEN)	Variable	Variable	At least 30% neuroendocrine component (Well or poorly differentiated) and non- neuroendocrine epithelial components (mostly adenocarcinoma)

Some site-specific IHC markers can help distinguish between NECB and metastatic NEC from lung or gastrointestinal sites. TTF-1 is usually positive in approximately 70% of lung metastases, while CDX-2 is positive in 80%–100% of gastrointestinal metastases, and both of them are usually negative in NECB. Previous reports indicated that positive TTF-1 expression can also be found in poorly differentiated mammary NECs. GATA3, mammaglobin, and GCDFP15 are usually positive for primary NECB and negative for secondary tumors. However, it is important to emphasize that GATA-3 is non-specific for NECB and has been reported in other cancers such as urothelial carcinomas, mesotheliomas, squamous cell carcinomas, and renal epithelial tumors. Regarding molecular characteristics of NECB, they are usually hormone receptor-positive (ER/PR +ve) and human epidermal growth factor receptor 2 (HER-2)-negative. There are only very few data regarding mutational profile of NECB. Previous results demonstrated PIK3CA mutation in 7%–20%, GATA-3, FOXA1, TBX3, and ARID1A mutations in approximately 17%, and low frequency of AKT1 and CDH1 mutations. However, TP53 and Rb1 loss is frequent in NEC, almost all the data exclusively are for GI-NEC and few data showed that NECB has a markedly low rate of these mutations (<7%) [15]. Regarding diagnostic imaging for NECB, work up with computed tomography (CT) scan of the chest and abdomen should be performed to look for possible primary tumors with breast metastasis. Positron emission tomography scan (PET-FDG 18) provides additional diagnostic clues when the discrimination of primary and secondary NEC remains doubtful. Some practical criteria can be used for differential diagnosis. For example, masses with absence of *in situ* involvement, negative ER/PR, and the absence of involved axillary lymph nodes are highly indicative of a secondary rather than a primary tumor [16].

## Treatment

### Localized Disease

A minority of patients with a NECB will present with localized disease but, given its rarity, there are no randomized trials available to guide management. This is important to note, as such a trial could potentially guide the treatment of NECB and compare combination regimens to ductal carcinoma. The therapeutic approach for localized disease is not based on prospective data but rather based primarily on retrospective studies and case reports. Surgical excision is the preferred option for localized disease, similar to ductal and lobular breast cancer. As in other types of breast cancer, adjuvant chemotherapy and/or chemoradiation is employed for NEBC after curative intent surgery depending on tumor size, lymph node involvement, and metastasis. There is no sufficient data that have compared the benefit from adjuvant platinum-based versus taxane-based and/or anthracycline chemotherapy regimens. Long-term survival benefit for etoposide plus cisplatin or carboplatin (EP) adjuvant chemotherapy for NEC has been extrapolated from SCLC, and no study has included patients with NECB. Regarding the optimal number of cycles in the adjuvant setting there is not enough data to support six cycles over four cycles of EP. Therefore, the number of adjuvant chemotherapy cycles should be based on patient tolerability.

Data for using taxane-based or TEC (docetaxel, epirubicin, and cyclophosphamide) chemotherapy in NECB has been associated with partial response mainly in the neoadjuvant setting in case reports and retrospective analysis [17]. There is limited data to consider these regimens for the adjuvant setting. Therefore, it should be reserved for patients with inoperable or large tumors in which breast-conserving surgery has been selected. The survival benefit of adding concurrent or sequential radiation to EP chemotherapy in NECB is based upon data from limited-stage SCLC, and there are no specific randomized trials for EP-NEC. Consequently, adjuvant chemo-radiation should be considered in selected patients with locally advanced disease (T3-T4, positive surgical margins, and/or lymph node involvement). Given that most patients with NECB have positive ER/PR receptors, then considering endocrine therapy may be helpful. Case reports have mainly demonstrated significant response using neoadjuvant goserelin and letrozole in young patients with localized large (≥5 cm) poorly differentiated breast NEC. This suggests that neoadjuvant endocrine therapy may be considered for NECB, especially for patients with large tumors and positive ER/PR receptors. However, HER-2 amplification has been reported in NECB, data for using HER2 monoclonal antibody therapy has been limited to case reports in adjuvant settings, and there is not enough evidence to combine it with current adjuvant cytotoxic chemotherapy [17–28] (Table 2).

**TABLE 2:** Data from case reports and retrospective studies for localized NECB [1–12].

Author	Stage	NEC subtype	Ki67%	Hormone status	Surgery	Neoadjuvant chemotherapy	Adjuvant chemotherapy	Hormonal therapy
Christie [20]	IIIC	SC	NR	ER/PR/Her-2 −ve	Yes	No	Yes	No
							CBDCA/VP-16	
Nicoletti [21]	IIB	SC	90%	ER/PR +ve	Yes	No	Yes	Yes
				Her-2 −ve			AC+CBDCA/VP-16	
Latif [22]	IIB	SC	NR	ER/PR/Her-2 −ve	Yes	No	Ys	No
							CBDCA/VP-16	
Yildirim [23]	IIIB	NR	60%	ER/PR/Her-2 −ve	Yes	No	Yes	No
							CDDP/VP-16	
Yildirim [23]	IIIB	NR	50%	ER/PR +ve	Yes	No	Yes	Yes
				Her-2 −ve			CDDP/VP-16	
Watrowski [28]	IA	MiNEN	46%	ER/PR +ve	Yes	No	Yes	Yes
				Her-2 −ve			CDDP/VP-16	
Angarita [19]	IIIB	NR	>20%	ER+ve	Yes	Yes	Yes	Yes
				PR/Her-2 −ve		Doxorubicin and cyclophosphamide	CDDP/VP-16	
Pagano [24]	IIIA	NR	30%	ER/PR +ve	Yes	No	Yes	Yes
				Her-2 −ve			CMF	
Wei [17]	IIIA	NR	40%	ER+ve	Yes	Yes	Yes	No
				PR/Her-2 −ve			EC + DTX	
Janosky [25]	IIA	LC	100%	ER/PR/Her-2 −ve	Yes	Yes	Yes	No
						Doxorubicin, and cyclophosphamide followed by paclitaxel	AC + DTX	
Abou Dalle [18]	IIA	SC	50%	ER/Her-2 −ve	Yes	No	Yes	No
				PR +ve			CDDP/VP-16 + FEC	
Valente [26]	IIB	NR	90%	ER/PR +ve	Yes	No	Yes	Yes
				Her-2 −ve			FEC	
Kawasaki [27]	IIIA	LC	75%	ER/PR/Her-2 −ve	Yes	No	Yes	No
							EC	

SC, small cell; LC, large cell; MiNEN, Mixed Non-neuroendocrine-neuroendocrine neoplasm; ER, estrogen receptor; PR, progesterone receptor; HER2, human epidermal growth factor receptor 2; ET, endocrine therapy; Chemo, chemotherapy; NR, non-reported. CHEMO: AC, Adriamycin (Doxorubicin)/Cyclophosphamide; CBDCA, carboplatin; CDDP, cisplatin; DTX, docetaxel; EC, Epirubicin/Cyclophosphamide; FEC, Fluorouracil/Epirubicin/Cyclophosphamide; VP-16, Etoposide.

### Metastatic Disease

**FIGURE 3:**
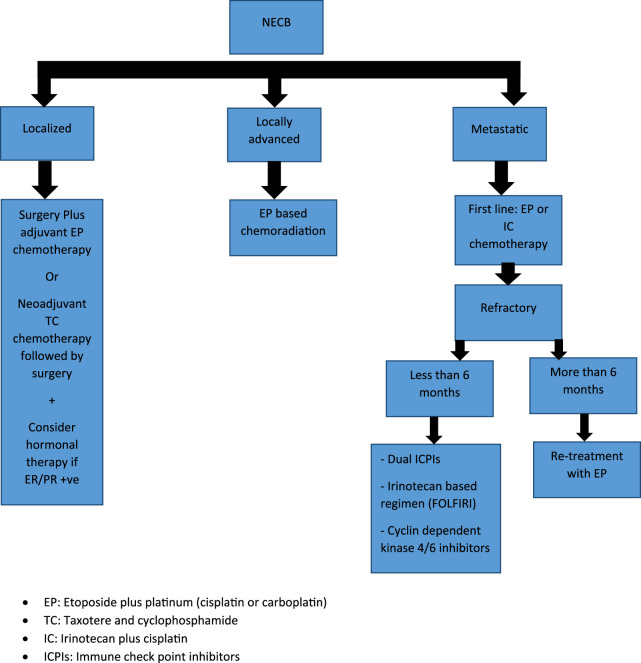
Treatment algorithm for Neuroendocrine Breast Carcinoma (NEBC): EP, Etoposide plus platinum (cisplatin or carboplatin); TC, Taxotere and cyclophosphamide; IC, Irinotecan plus cisplatin; ICPIs, Immune check point inhibitors.

Palliative systemic chemotherapy is the main treatment of patients with metastatic NEC. First line therapy for NEC has been historically adapted from small cell lung cancer (SCLC), with etoposide plus platinum (Cisplatin or Carboplatin) as the standard regimen. The data was primarily retrospective with an RR range between 40% and 50%, PFS of 6 months, and median OS between 10 and 14 months with 2-year SR of <11%. A recently published phase III study (JCOG-1213 trial) evaluated irinotecan-based regimens (irinotecan plus cisplatin doublet, IP) versus platinum etoposide (EP) in 170 EP-NEC patients. There were no significant differences in either PFS (5.6 months vs. 5.1 months, HR 1.060, 95% CI, 0.777–1.445) or objective response rates (54.5% vs. 52.5%) between EP and IP respectively. On the basis of this trial, we can conclude that either chemotherapy regimen could be considered as a first line option for metastatic EP-NEC. The previous retrospective analysis and recent prospective trials mainly included patients with gastrointestinal and hepatobiliary NEC, and none of them actually included NEBC. Although there are no clear data on the outcomes of these regimens in NECB, it is reasonable to utilize similar chemotherapy regimens given similar disease biology between NEBC and other EP-NEC. Most patients who progress after first line cytotoxic chemotherapy have limited therapeutic options due to lack of supportive data for standard of care. Second-line agents were studied in EP-NEC, such as 5-FU combined with oxalipltain or irinotecan (FOLFOX/FOLFIRI) and a temozolomide-based regimen is mainly used for GEP-NEC with no similar available data for NECB [29]. One study demonstrated improved progression-free survival rate (PFS) with temozolomide in a patient with refractory advanced NECB [25]. Another potential targeted therapy is the use of cyclin dependent kinase 4/6 inhibitors, e.g., palbociclib, ribociclib, or abemaciclib, as in a case report palbociclib in conjunction with endocrine therapy was shown to be associated with a dramatic response in a case of poorly-differentiated NECB with HR-positive/HER-2 negative expression, and can present a future target for this patient population [30]. Regarding the role of immunotherapy, current data from dual check point inhibitors (ICPIs) in EP-NEC are highly variable and there is no enough similar data ICPIs for NECB [31]. Dual ICPIs (ipilimumab plus nivolumab) can be considered for those who are lacking other therapeutic options depending on the phase II trials for EP-NEC (DART and CA209-538) especially for patients with high tumor mutational burden (TMB) or mismatch repair deficient (MSI-H). Considering previous data demonstrated that a single-agent ICPI is not an effective option for EP-NEC and should not be considered for refractory NECB. Adjuvant endocrine therapy should be considered for patients with hormone receptor-positive NECB [19] (Figure 3).

### Prognosis

The prognosis of NECB remains controversial. Several studies have been published with conflicting results, and this might be explained by the varying criteria included to identify NECB (WHO 2003 or 2012) and the limited number of cases and data. There is a consensus that when compared to invasive breast carcinoma, those with NECB have worse outcomes with shorter overall-survival (OS). Some data have suggested similar outcomes between small cell and large cell NECB, but another study has shown that small cell NECB has the worst prognosis.

## Conclusion

NECB is an extremely rare NEC subtype. Because of limited evidence, the diagnosis can be challenging, with overlap with other types of breast cancers and difficulty distinguishing primary from secondary tumors. Treatment for NECB is based on available data from other known NECs with the same pathogenesis and histological behavior, such as GEP-NEC and SCLC. Adjuvant therapy is mainly composed of etoposide and platinum-based regimens with/without radiation. Regimens for metastatic NECB are even more limited, with first line therapy being composed primarily of etoposide and platinum-based regimens and no clear data regarding treatment in a refractory setting. Therefore, more research is needed to further identify therapeutic targets and aid in creating more targeted treatments that can lead to improved outcomes in patients with NECB.

## References

[1] Aggarwal G Singh S Kataria SP Kalra R Duhan A Sen R. Primary Neuroendocrine Carcinoma of Breast. J Cytol (2011) 28(2):91–2. 10.4103/0970-9371.80755 21713100 PMC3111719

[2] Feyrter F Hartmann G. On the Carcinoid Growth Form of the Carcinoma Mammae, Especially the Carcinoma Solidum (Gelatinosum) Mammae. Frankf Z Pathol (1963) 73:24–39. 14097474

[3] Vogler E. Uber das Basilare Helle-Zellen-Organ der Menschlichen Brustdruse. Klinische Medizin; osterreichische Z wissenschaftliche praktische Medizin (1947) 2:159–68. 20244863

[4] Irshad A Ackerman SJ Pope TL Moses CK Rumboldt T Panzegrau B. Rare Breast Lesions: Correlation of Imaging and Histologic Features With WHO Classification. Radiographics (2008) 28(5):1399–414. 10.1148/rg.285075743 18794315

[5] Bocker W. WHO Classification of Breast Tumors and Tumors of the Female Genital Organs: Pathology and Genetics. Verh Dtsch Ges Pathol (2002) 86:116–9. 12647359

[6] Trevisi E La Salvia A Daniele L Brizzi MP De Rosa G Scagliotti GV Neuroendocrine Breast Carcinoma: A Rare But Challenging Entity. Med Oncol (2020) 37(8):70. 10.1007/s12032-020-01396-4 32712767 PMC7382662

[7] Tan PH Ellis I Allison K Brogi E Fox SB Lakhani S The 2019 World Health Organization Classification of Tumours of the Breast. Histopathology (2020) 77(2):181–5. 10.1111/his.14091 32056259

[8] Wei B Ding T Xing Y Wei W Tian Z Tang F Invasive Neuroendocrine Carcinoma of the Breast: A Distinctive Subtype of Aggressive Mammary Carcinoma. Cancer (2010) 116(19):4463–73. 10.1002/cncr.25352 20572042

[9] Wang J Wei B Albarracin CT Hu J Abraham SC Wu Y. Invasive Neuroendocrine Carcinoma of the Breast: A Population-Based Study From the Surveillance, Epidemiology and End Results (SEER) Database. BMC Cancer (2014) 14:147. 10.1186/1471-2407-14-147 24589259 PMC3974013

[10] Woodard BH Eisenbarth G Wallace NR McCarty KS Mossler JA. Adrenocorticotropin Production by a Mammary Carcinoma. Cancer (1981) 47(7):1823–7. 10.1002/1097-0142(19810401)47:7<1823::aid-cncr2820470717>3.0.co;2-2 6261928

[11] Kaneko H Hojo H Ishikawa S Yamanouchi H Sumida T Saito R. Norepinephrine-Producing Tumors of Bilateral Breasts: A Case Report. Cancer (1978) 41(5):2002–7. 10.1002/1097-0142(197805)41:5<2002::aid-cncr2820410547>3.0.co;2-2 647636

[12] Coombes RC Easty GC Detre SI Hillyard CJ Stevens U Girgis SI Secretion of Immunoreactive Calcitonin by Human Breast Carcinomas. Br Med J (1975) 4(5990):197–9. 10.1136/bmj.4.5990.197 1191996 PMC1675000

[13] Ogawa H Nishio A Satake H Naganawa S Imai T Sawaki M Neuroendocrine Tumor in the Breast. Radiat Med (2008) 26(1):28–32. 10.1007/s11604-007-0182-y 18236131

[14] Tremelling A Samuel S Murray M. Primary Small Cell Neuroendocrine Carcinoma of the Breast - A Case Report and Review of the Literature. Int J Surg Case Rep (2017) 38:29–31. 10.1016/j.ijscr.2017.07.002 28734185 PMC5521029

[15] Lavigne M Menet E Tille JC Lae M Fuhrmann L Bonneau C Comprehensive Clinical and Molecular Analyses of Neuroendocrine Carcinomas of the Breast. Mod Pathol (2018) 31(1):68–82. 10.1038/modpathol.2017.107 28884749

[16] Shetty MR. Neuroendocrine Primary Small Cell Carcinoma of the Breast. Am J Clin Oncol (1996) 19(3):322–3. 10.1097/00000421-199606000-00024 8638551

[17] Wei X Chen C Xi D Bai J Huang W Rong L A Case of Primary Neuroendocrine Breast Carcinoma That Responded to Neo-Adjuvant Chemotherapy. Front Med (2015) 9(1):112–6. 10.1007/s11684-014-0345-z 25098433

[18] Abou Dalle I Abbas J Boulos F Salem Z Assi HI. Primary Small Cell Carcinoma of the Breast: A Case Report. J Med Case Rep (2017) 11(1):290. 10.1186/s13256-017-1467-0 29047418 PMC5648460

[19] Angarita FA Rodríguez JL Meek E Sánchez JO Tawil M Torregrosa L. Locally-Advanced Primary Neuroendocrine Carcinoma of the Breast: Case Report and Review of the Literature. World J Surg Oncol (2013) 11(1):128. 10.1186/1477-7819-11-128 23734899 PMC3682896

[20] Christie M Chin-Lenn L Watts MM Tsui AE Buchanan MR. Primary Small Cell Carcinoma of the Breast With TTF-1 and Neuroendocrine Marker Expressing Carcinoma *In Situ* . Int J Clin Exp Pathol (2010) 3(6):629–33. 20661411 PMC2907125

[21] Nicoletti S Papi M Drudi F Fantini M Canuti D Tamburini E Small Cell Neuroendocrine Tumor of the Breast in a 40 Year-Old Woman: A Case Report. J Med Case Rep (2010) 4:201. 10.1186/1752-1947-4-201 20591162 PMC2913980

[22] Latif N Rosa M Samian L Rana F. An Unusual Case of Primary Small Cell Neuroendocrine Carcinoma of the Breast. Breast J (2010) 16(6):647–51. 10.1111/j.1524-4741.2010.00974.x 21070442

[23] Yildirim Y Elagoz S Koyuncu A Aydin C Karadayi K. Management of Neuroendocrine Carcinomas of the Breast: A Rare Entity. Oncol Lett (2011) 2(5):887–90. 10.3892/ol.2011.320 22866145 PMC3408058

[24] Pagano M Asensio SN Zanelli F Lococo F Cavazza A Damiani S Is There a Role for Hormonal Therapy in Neuroendocrine Carcinoma of the Breast? A Paradigmatic Case Report. Clin Breast Cancer (2014) 14(5):e99–e101. 10.1016/j.clbc.2014.03.001 24958323

[25] Janosky M Bian J Dhage S Levine J Silverman J Jors K Primary Large Cell Neuroendocrine Carcinoma of the Breast, a Case Report With an Unusual Clinical Course. Breast J (2015) 21(3):303–7. 10.1111/tbj.12403 25823996

[26] Valente I Tringali G Martella EM Pallavera L D'Aloia C. Primary Neuroendocrine Carcinoma of the Breast: A Case Report of Liver and Lymph Node Metastases After Eight Years From Diagnosis. Breast J (2020) 26(3):505–7. 10.1111/tbj.13535 31513314

[27] Kawasaki T Hasebe T Oiwa M Sugiyama K Muramatsu C Ueda S Invasive Carcinoma With Neuroendocrine Differentiation of the Breast Showing Triple Negative, Large and Basal Cell-Like Features. Pathol Int (2019) 69(8):502–4. 10.1111/pin.12832 31338942

[28] Watrowski R Jager C Mattern D Horst C. Neuroendocrine Carcinoma of the Breast--Diagnostic and Clinical Implications. Anticancer Res (2012) 32(11):5079–82. 23155283

[29] Lamarca A Frizziero M Barriuso J McNamara MG Hubner RA Valle JW. Urgent Need for Consensus: International Survey of Clinical Practice Exploring Use of Platinum-Etoposide Chemotherapy for Advanced Extra-Pulmonary High Grade Neuroendocrine Carcinoma (EP-G3-NEC). Clin Translational Oncol (2019) 21(7):950–3. 10.1007/s12094-018-1996-z 30506132

[30] Shanks A Choi J Karur V. Dramatic Response to Cyclin D-Dependent Kinase 4/6 Inhibitor in Refractory Poorly Differentiated Neuroendocrine Carcinoma of the Breast. Baylor Univ Med Cent Proc (2018) 31(3):352–4. 10.1080/08998280.2018.1463041 PMC5997053 29904310

[31] Vranic S Palazzo J Sanati S Florento E Contreras E Xiu J Potential Novel Therapy Targets in Neuroendocrine Carcinomas of the Breast. Clin Breast Cancer (2019) 19(2):131–6. 10.1016/j.clbc.2018.09.001 30268765

